# Efficacy and Safety of Low‐Dose Spironolactone in Management of Diabetic Kidney Disease in a Real‐World Setting

**DOI:** 10.1002/edm2.70058

**Published:** 2025-06-05

**Authors:** Manochehr Amini, Davood Dalil, Fatemeh Yaghoubi, Zoleykha Valizadeh, Farnaz Tavakoli, Zohreh Koohpayezadeh

**Affiliations:** ^1^ Department of Internal Medicine, Shariati Hospital, School of Medicine Tehran University of Medical Sciences Tehran Iran; ^2^ School of Medicine Shahed University Tehran Iran; ^3^ Nephrology Research Center, Shariati Hospital Tehran University of Medical Sciences Tehran Iran

**Keywords:** albuminuria, diabetic kidney disease, diabetic nephropathy, mineralocorticoid receptor antagonists (MRA), spironolactone

## Abstract

**Background:**

Adding spironolactone to renin‐angiotensin‐aldosterone system (RAAS) blockers has been shown to reduce albuminuria in patients with diabetic kidney disease (DKD). However, the increased risk of hyperkalaemia is a major concern. This study aimed to evaluate the efficacy and safety of low‐dose (12.5 mg/day) spironolactone in reducing albuminuria and improving renal function in Iranian adults with DKD.

**Methods:**

This was a pre‐post‐treatment study of 60 patients with type 2 diabetes, age > 18 years, albuminuria ≥ 30 mg, estimated glomerular filtration rate (eGFR) ≥ 30 mL/min/1.73 m^2^, and serum potassium level ≤ 5 mEq/L, who were referred to the diabetes clinic of Shariati Hospital, Tehran, Iran. The patients were prescribed spironolactone (12.5 mg) once daily. Changes in urinary albumin excretion (U.Alb), urine albumin‐to‐creatinine ratio (uACR), blood pressure, serum creatinine (Cr) levels, and serum potassium levels from the baseline over the 12‐week intervention period were measured. Statistical significance was set at *p* < 0.05.

**Results:**

After 12 weeks of taking 12.5 mg/day spironolactone, there was a statistically significant but modest increase in eGFR (*p* = 0.042), and a statistically significant decrease was observed in both the U.Alb and uACR (*p* < 0.001). There was a significant reduction in the mean Cr level (*p* = 0.003). Systolic blood pressure did not decrease significantly (*p* = 0.079), but diastolic blood pressure decreased significantly (*p* = 0.007). Changes in serum potassium levels over time were not significant (*p* = 0.302). The reduction in albuminuria in patients taking only spironolactone and those taking spironolactone with SGLT2i was not significantly different (*p* = 0.916 and *p* = 0.948, respectively).

**Conclusions:**

This study found that adding spironolactone to RAAS blockers effectively reduced albuminuria, mildly increased eGFR, and improved renal outcomes in patients with DKD. Additionally, spironolactone significantly reduced albuminuria, regardless of the concurrent use of SGLT2i.

## Graphical abstract

The pre‐post‐treatment study of 60 patients with diabetic kidney disease (DKD) showed that adding 12.5 mg/day spironolactone to RAAS blockers effectively reduced albuminuria, mildly increased eGFR, and improved renal outcomes. Additionally, spironolactone significantly reduced albuminuria, regardless of concurrent use of SGLT2i.

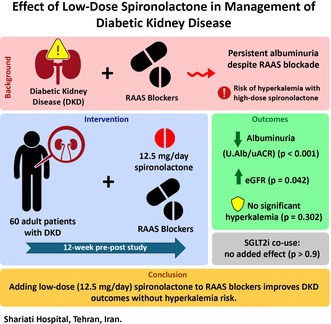

**Summary**

- Although previous studies showed that adding 25–100 mg/day spironolactone to ACEI or ARB reduced albuminuria in DKD patients, hyperkalaemia and hypotension were reported as side effects.
- The study showed that the addition of low‐dose (12.5 mg/day) spironolactone to ACEI or ARB in patients with DKD was an effective and safe treatment to significantly reduce albuminuria, increase eGFR, and improve renal outcomes without causing hyperkalaemia.
- In addition, we showed that before and after 12 weeks of spironolactone use, there was no significant difference in the level of albuminuria between patients taking SGLT2i and those not taking it.

## 1 Introduction

Diabetic kidney disease (DKD) is a leading cause of chronic kidney disease (CKD), followed by end‐stage renal disease (ESRD), affecting well over 700 million individuals worldwide [1, 2]. It affects 30% of patients with type 1 (T1DM) and 40% of patients with type 2 (T2DM) diabetes [3]. DKD refers to persistent albuminuria or decreased estimated glomerular filtration rate (eGFR) in patients with diabetes, without implying specific underlying kidney pathology [4]. It could be classified based on the presence of proteinuria, the classical type known as diabetic nephropathy (DN), and the non‐classical type identified with decreased eGFR without proteinuria, known as nonproteinuric DKD (NP‐DKD) [5, 6].

Blocking the renin‐angiotensin‐aldosterone system (RAAS) using angiotensin‐converting enzyme inhibitors (ACEIs) and angiotensin receptor blockers (ARBs) has been shown to significantly slow the progression of kidney disease [5, 7]. However, some patients with DN, particularly those who have used ACEIs or ARBs for extended periods, experience a return of aldosterone to its previous levels due to the aldosterone escape phenomenon [8]. Aldosterone can cause nephrosclerosis and kidney fibrosis in patients with DN and hypertension [7]. Moreover, studies have indicated that the use of ACEI and ARB alone cannot prevent the effects of aldosterone. Therefore, adjuvant treatment with mineralocorticoid receptor antagonists such as spironolactone to improve proteinuria in patients with DN is justified [5, 9].

Studies have shown that adding spironolactone to RAAS blockers is more effective in reducing albuminuria in DKD patients [8, 10, 11]. In addition, the low price of spironolactone in the market is one of the most important features of this drug, especially in developing countries, compared with other newly established drugs that effectively reduce proteinuria, such as sodium‐glucose cotransporter‐2 inhibitors (SGLT2is). However, the increased risk of hyperkalaemia following the administration of spironolactone is a major concern, making physicians hesitant to prescribe it to such patients [10]. Many patients with DN already have underlying hyperkalaemia due to type 4 renal tubular acidosis (RTA). Administration of aldosterone antagonists can worsen hyperkalaemia in these patients [12]. In previous studies, spironolactone was prescribed at a dose of 25–100 mg/day [10, 11].

Therefore, this study aimed to evaluate the efficacy and safety of a lower dose (12.5 mg/day) of spironolactone in lowering albuminuria and improving renal function in Iranian adults with DN. Because of known risks of hyperkalaemia with higher doses, we specifically evaluated whether a reduced dose would preserve clinical benefit without increasing adverse effects. Since spironolactone is low‐cost and readily available, proof of its efficacy and safety with a lower dose might be a feasible, low‐cost option for enhancing renal outcomes in DKD. Additionally, we examined whether low‐dose add‐on therapy with spironolactone might have additive effects when combined with conventional RAAS blockers, with or without SGLT2is.

## 2 Materials and Methods

### 2.1 Study Settings and Participants

The current investigation is a non‐randomised interventional (pre‐ and post‐treatment) study of patients with DN referred to the diabetes clinic of Shariati Hospital, Tehran, Iran. This study selected and included 60 patients. Inclusion criteria were as follows: patients with T2DM, age older than 18 years, albuminuria ≥ 30 mg despite receiving at least 8 weeks of ARB or ACEI drugs, eGFR ≥ 30 mL/min/1.73 m^2^, serum potassium (K) level ≤ 5 meq/L, and systolic blood pressure (BP) above 100 mmHg. People who do not meet the requirements are those whose haemoglobin A1c (HbA1C) level is 8.5 or higher, who have taken NSAIDs or immunosuppressant drugs in the past, who have an autoimmune disease that causes albuminuria, who are pregnant or breastfeeding, who are already taking spironolactone, who have a history of drug sensitivity, or who refuse to give informed consent to participate in the study.

For every subject throughout the course of this 12‐week study, baseline medications such as SGLT2is, ACEIs, ARBs, and other antidiabetic medications were continued without any adjustments. Dose modifications or new medication additions were only prohibited unless they were medically necessary, whereupon the patient would be withdrawn from this study.

### 2.2 Data Gathering

After explaining the study aims and plan, demographic data such as age, sex, history of diabetes, smoking habits, use of ARB or ACEI, use of SGLT2i, and type of diabetes medications were recorded for each patient. The patients were prescribed spironolactone at a dose of 12.5 mg once daily. We provided them with detailed information about the common side effects of the drug, including hypotension and hyperkalaemia. BP, fasting blood sugar (FBS) level, HbA1C, serum K level, serum creatinine (Cr) level, level of urinary albumin (U.Alb), and urine albumin‐to‐creatinine ratio (uACR) were all measured twice: at the beginning of the study and 12 weeks after using spironolactone (the end point of the study). An internist or nephrologist followed up and visited the patients five times during the study period, at weeks 0, 2, 4, 6, and 12. At each visit, the patient's serum K level was also measured. All measurements were conducted at the laboratory of Shariati Hospital, Tehran, Iran, under standardised conditions and methods. If any patients experienced side effects such as hypotension, hyperkalaemia (*K* > 5), or drug intolerance, they were excluded from the study, and appropriate measures were taken.

### 2.3 Statistical Analysis

Data were analysed using SPSS software (version 26.0). Descriptive statistics were used to summarise the demographic and clinical characteristics of the study population. After evaluating the normality distribution of data, the paired *t*‐test or Wilcoxon signed‐rank test was used to compare the changes of variables with their baseline values. The repeated measures one‐way ANOVA was used to investigate the changes of serum K over time during the study period. *p* < 0.05 were considered statistically significant.

## 3 Results

Table 1 displays the baseline characteristics of patients in this study. This study included 60 patients with DKD, whose ages ranged from 38 to 75 years. 26 (43.33%) were female and 34 (56.67%) were male.

**TABLE 1:** Baseline characteristics of the study population.

Variable	*N* (%)/mean ± SD
Age (years)	56.47 ± 11.22
≥ 65 years	16 (27.7)
< 65 years	44 (73.3)
Gender	
Male	34 (56.67)
Female	26 (43.33)
HxD (years)	10.77 ± 4.16
≥ 10 years	32 (53.3)
< 10 years	28 (46.7)
SBP (mmHg)	122.12 ± 10.43
DBP (mmHg)	78.59 ± 5.61
eGFR (mL/min/1.73 m^2^)	71.83 ± 19.09
Variable	N (%) / mean ± SD
Medication	
ACEI	8 (13.3)
ARB	52 (86.7)
SGLT2i	20 (33.3)
Metformin	49 (81.7)
Linagliptin	16 (26.7)
Gliclazide	3 (5.0)
FBS (mg/dayL)	129.17 ± 12.67
HbA1C	7.07 ± 0.68
Cr (mg/dayL)	1.08 ± 0.23
U. Alb (mg)	321.10 ± 476.48
uACR (mg/g)	254.61 ± 382.42
K (meq/L)	4.26 ± 0.24

Abbreviations: Cr, creatinine; DBP, diastolic blood pressure; eGFR, estimated glomerular filtration rate; FBS, fasting blood sugar; HxD, history of diabetes; K, potassium; SBP, systolic blood pressure; U.Alb, urinary albumin; uACR, urine albumin‐to‐creatinine ratio.

Table 2 demonstrates the changes in kidney function parameters, including serum Cr, eGFR, U.Alb, and uACR. We used the Wilcoxon signed‐rank test to look at the changes in the parameters. After 12 weeks of taking spironolactone, there was a statistically significant rise in mean eGFR (*p* = 0.042) and big drops in U.Alb and uACR (*p* < 0.001). Furthermore, there was a significant reduction in the mean level of Cr after 3 months compared to the beginning of the study (*p* = 0.003). Although the systolic BP reduced over the study period, the changes were not statistically significant. However, the mean diastolic BP showed a statistically significant decrease after 3 months.

**TABLE 2:** Changes in the renal outcomes during 12 weeks of treatment with 12.5 mg/day spironolactone.

Variable	Baseline^b^	End point^b^	*p* ^a^
SBP (mmHg)	122.12 ± 10.43	119.97 ± 10.63	0.079
DBP (mmHg)	78.59 ± 5.61	76.17 ± 4.99	0.007
Cr (mg/dL)	1.08 ± 0.23	1.01 ± 0.23	0.003
eGFR (mL/min/1.73 m^2^)	71.83 ± 19.09	74.25 ± 17.81	0.042
U.Alb (mg)	321.10 ± 476.48	248.71 ± 293.44	< 0.001
uACR (mg/g)	254.61 ± 382.42	203.06 ± 267.15	< 0.001

Abbreviations: Cr, serum creatinine; DBP, diastolic blood pressure; eGFR, estimated glomerular filtration rate; SBP, systolic blood pressure; U.Alb, urinary albumin; uACR, urine albumin‐to‐creatinine ratio.

^a^ Analysed by the Wilcoxon signed‐rank test.

^b^ Mean ± SD.

**FIGURE 1:**
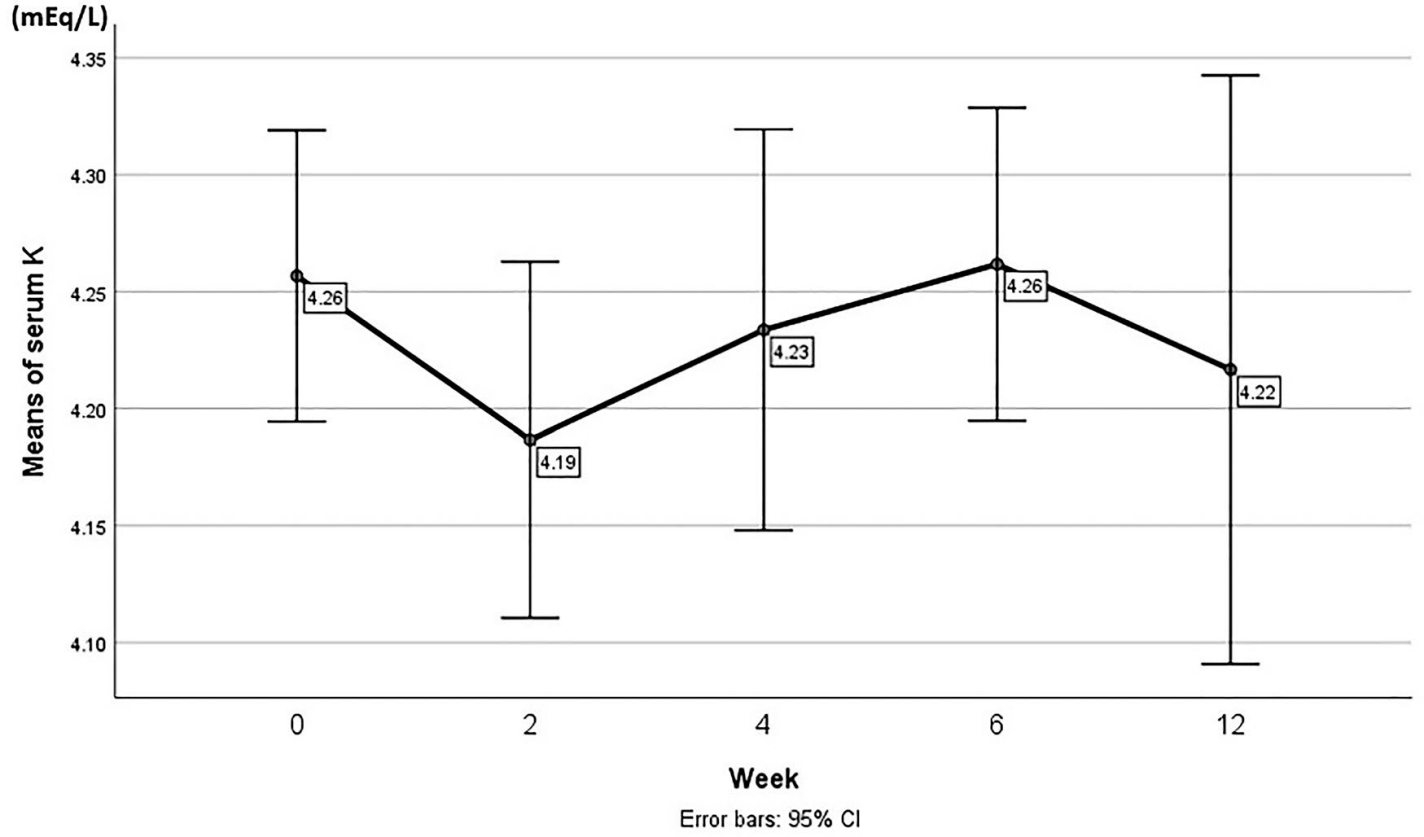
Mean serum potassium (K^+^) levels (± standard deviation) measured at baseline and at weeks 2, 4, 6, and 12 during treatment with 12.5 mg/day spironolactone. Error bars represent 95% confidence intervals. All patients (*n* = 60) were evaluated at each time point. No significant changes in potassium levels were observed over time (*p* = 0.302).

Regarding the potential risk of hyperkalaemia during long‐term use of spironolactone, the serum K levels were measured at weeks 0, 2, 4, 6, and 12. One‐way ANOVA analysis showed that the mean serum K changes over time were not significant (*p* = 0.302). Figure 1 demonstrates the changes in serum K levels over the 12 weeks of using low‐dose spironolactone.

In subgroup analyses, proteinuria levels in patients treated with spironolactone plus a RAAS blocker were compared to those receiving spironolactone in conjunction with SGLT2i and a RAAS blocker (Table 3). The findings indicated a notable reduction in the mean levels of U.Alb and uACR after 3 months of spironolactone treatment in both groups (*p* < 0.05). The Mann–Whitney *U* test was employed to assess albuminuria between the two groups, those without SGLT2i and those with SGLT2i, both prior to and following spironolactone administration, revealing no significant differences (*p* > 0.05). The levels of U.Alb and uACR over time in patients taking only spironolactone and those taking spironolactone in conjunction with SGLT2i were not significantly different (*p* = 0.916, *p* = 0.948, respectively).

**TABLE 3:** Comparison of the outcomes at the beginning and end of the study (after 3 months).

Variable	Group	Baseline^c^	End point^c^	*p* ^a^
U.Alb (mg)	Without SGLT2i	273.82 ± 319.51	242.90 ± 268.94	< 0.001
	With SGLT2i	415.65 ± 693.79	260.95 ± 347.17	0.001
	*p* ^b^	0.981	0.916	
uACR (mg/g)	Without SGLT2i	224.67 ± 291.99	207.82 ± 270.73	< 0.001
	With SGLT2i	314.50 ± 522.91	193.05 ± 266.49	0.001
	*p* ^b^	0.712	0.948	

Abbreviations: SGLT2i, sodium‐glucose cotransporter‐2 inhibitor; U.Alb, Urinary albumin; uACR, Urine albumin‐to‐creatinine ratio.

^a^ Analysed by the Wilcoxon signed‐rank test.

^b^ Analysed by the Mann–Whitney *U* test.

^c^ Mean ± SD.

## 4 Discussion

The findings of this study demonstrate that adding daily low‐dose spironolactone (12.5 mg) to RAAS blockers significantly reduces albuminuria and improves renal outcomes in patients with DKD. Interestingly, this was accomplished with minimal risk of hyperkalaemia, a constant fear with using spironolactone. The clinical utility of this strategy is most pertinent in low‐ and middle‐income countries, where access to and affordability of newer renoprotective therapies like SGLT2is are uncertain. Our results indicate that low‐dose spironolactone (12.5 mg/day) can be a low‐cost option or adjunct therapy in the management of DKD.

Previously, the anti‐albuminuric effect of spironolactone was well documented. However, most studies used 25–100 mg/day spironolactone. Hou et al.'s study in China showed that spironolactone at a dose of 25 mg/day in addition to ARB therapy resulted in a dramatic reduction in proteinuria in patients with DKD, although this was at the expense of an increased frequency of hyperkalaemia [10]. A multi‐centre RCT of 52 Japanese DN patients by Kato et al. showed the anti‐albuminuric effect of 25 mg/day spironolactone along with conventional RAAS inhibitors for 8 weeks [13]. In a meta‐analysis study of 640 patients with hypertension and diabetes, Lin et al. showed that compared to placebo, 25–50 mg/day of spironolactone significantly reduced BP and U.Alb but increased serum Cr. They noted a 2.5% incidence of mild to moderate hyperkalaemia and a 1.6% incidence of severe hyperkalaemia [14]. Oxlund et al., in a study of 112 adults with resistant hypertension and T2DM, revealed that 25 mg/day spironolactone induces a significant reduction in BP and uACR [15].

In a clinical trial of 59 patients with T2DM and macroalbuminuria, van den Meiracker et al. showed that adding 25–50 mg spironolactone to an ACEI or ARB compared with placebo reduced albuminuria by 40.6%, increased serum Cr levels, and decreased eGFR, likely due to higher doses and more advanced kidney disease in their cohort [16]. Similarly, an RCT of 60 patients by Ziaee et al. demonstrated that adding 25 mg/day spironolactone to 50 mg/day enalapril compared to taking enalapril alone significantly reduced albuminuria without showing any significant adverse effect, including hyperkalaemia and hypotension [17].

In contrast, our study demonstrated a 20% reduction in albuminuria (uACR: 255 mg/g to 203 mg/g) with a lower dose of spironolactone (12.5 mg/day), without significant changes in K levels or renal function. This indicates that, although the reduction in albuminuria with 12.5 mg/day spironolactone is less notable, the lack of hyperkalaemia and other side effects renders it a safer choice for patients with DKD, particularly those with preserved renal function.

Before, three studies investigated the effect of administering 12.5 mg/day spironolactone on albuminuria in different settings. In 2009, Bomback et al. conducted a pre‐post study of 21 obese adults and found that the addition of 12.5 mg/day of spironolactone to an ACEI reduced BP and U.Alb [18]. Another study of 42 individuals with glomerulonephritis showed that adding 12.5 mg/day of spironolactone to ARB decreased proteinuria. Similar to our results, Oiwa et al., in a 24‐week randomised clinical trial of 130 Japanese patients with T2DM and CKD, demonstrated that low‐dose (12.5 mg/day) spironolactone significantly reduced uACR and BP without causing hyperkalaemia [19].

Regarding hyperkalaemia during spironolactone use, almost all studies conducted with 25–100 mg of spironolactone have reported some rate of hyperkalaemia among the study population [10, 14, 15, 16, 20, 21]. Lin et al. reported 2.5% mild to moderate and 1.6% severe hyperkalaemia among 640 patients [14]. Oxlunda et al. reported hyperkalaemia as the most frequent complication, leading to dose reduction in three patients and discontinuation in one [15]. In a study by Tofte et al. involving 1775 participants, 25 mg of spironolactone was used to assess the progression of microalbuminuria. The study reported that 13% of the participants using spironolactone experienced hyperkalaemia [20]. However, our study, similar to the Oiwa et al. study [19], showed that low‐dose (12.5 mg/day) spironolactone played a significant role in reducing albuminuria and increasing eGFR, without causing hyperkalaemia (*K* > 5 mEq/L) in any of the participants.

Although a limited number of studies have tested 12.5 mg/day spironolactone, our study contributes value by validating the efficacy and safety of this regimen in real‐world clinic‐based care in the Middle East (Iran). Unlike in well‐controlled RCTs, our patients were treated in routine clinical care with a wider array of comorbid illnesses and access to healthcare constraints. This environment approximates where spironolactone cost‐effectiveness would be seen to have the most impact. Additionally, the study involved serial K checks, proving it is feasible to utilise low‐dose spironolactone safely in clinic‐based care. Therefore, although the efficacy signal is consistent with prior data, the study yields useful evidence on implementation and real‐world applicability, notably in areas with reduced healthcare resources where hyperkalaemia concerns would restrict utilisation of spironolactone.

SGLT2is are glucose‐lowering drugs used to manage T2DM owing to their promising renal and cardiovascular protective effects. Recent studies have shown they reduce albuminuria and preserve eGFR in mild to moderate DKDs with eGFR 30–60 [22, 23, 24]. Recently, Vert et al. revealed that the combination of SGLT2i and RAAS blocker significantly improves prognosis in CKD patients without diabetes. However, the high cost of this drug, particularly in developing countries, is a major concern for patients. Spironolactone is inexpensive [25, 26, 27]. Therefore, in the subgroup analyses, we compared a triple therapy consisting of an SGLT2i, a RAAS blocker, and low‐dose spironolactone against a combination of low‐dose spironolactone and a RAAS blocker.

In this study, at the beginning and before using spironolactone, there was no significant difference in the level of albuminuria between patients who were taking SGLT2i and those who were not. In addition, after adding spironolactone to the treatment regimen in both groups, there was still no significant difference in the level of albuminuria between these two groups. Nevertheless, the addition of 12.5 mg/day spironolactone significantly reduced albuminuria in both groups. It should be noted that this secondary analysis was exploratory and was neither designed nor powered to test interaction effects. Therefore, such findings must be viewed cautiously and are not meant to alter the study's main purpose, which is focused on low‐dose spironolactone's independent effect.

This study faced several limitations. Firstly, the limited number of participants hindered the broad applicability of findings, particularly concerning side effects like hyperkalaemia. Secondly, the study employed a single‐arm, pre‐post interventional design that lacked a control group. The absence of randomization and blinding heightens the risk of bias and restricts our capacity to ascribe the observed changes exclusively to the intervention. Confounding factors such as the natural progression of disease, regression to the mean, or other unmeasured variables may have impacted the outcomes. Thirdly, since the study was carried out at a single centre and lasted only 12 weeks, the findings may not be applicable to wider populations or indicative of long‐term efficacy and safety. Finally, this investigation was not controlled for factors of diet, physical activity, or intake of sodium with the potential to influence albuminuria and renal function. Although these variables may introduce variability, all of the study participants were encouraged to continue their regular lifestyles throughout the study. Moreover, U.Alb measurements were conducted at different times in the outpatient clinic, which may have introduced variability associated with daily activities and dietary intake.

Regarding above limitations, our findings need to be tested in larger long‐term multi‐institution RCTs. These studies must have standardised medication adherence protocols, lifestyle variables (e.g., diet/exercise), and albuminuria measurement timing in order to improve generalisability. Dose–response analyses and hard renal outcome measures (i.e., progression to ESRD) also need to be examined in future studies. These are important steps to establish spironolactone's place in the management of DKD compared to newer agents such as SGLT2is.

## 5 Conclusion

This study showed that low‐dose spironolactone (12.5 mg/day) is an effective and safe treatment for reducing albuminuria and improving renal outcomes in patients with DKD, without inducing hyperkalaemia. In addition, we showed that before and after 12 weeks of spironolactone use, there was no significant difference in the level of albuminuria between patients taking SGLT2i and those not taking them. However, adding spironolactone to the treatment regimen of both groups significantly decreased the level of albuminuria. Although the reduction in albuminuria was small, the absence of significant side effects supports using 12.5 mg/day spironolactone as a viable option for patients with DKD, especially those at risk of hyperkalaemia. Future multicentre studies with larger populations must confirm these findings and explore the long‐term benefits of low‐dose spironolactone.

## Author Contributions

**Manochehr Amini**: Conceptualization; Methodology; Investigation; Writing – original draft. **Davood Dalil**: Data curation; Formal analysis; Visualization; Writing – original draft; Writing – review & editing. **Fatemeh Yaghoubi**: Supervision; Project administration; Conceptualization; Writing – review & editing. **Zoleykha Valizadeh**: Investigation; Data collection; Resources. **Farnaz Tavakoli**: Data collection; Validation; Writing – review & editing. **Zohreh Koohpayezadeh**: Data collection; Writing – review & editing. All authors have read and approved the final manuscript.

## Ethics Statement

This study was conducted ethically in accordance with the World Medical Association Declaration of Helsinki. The Research Ethics Committee of Tehran University of Medical Sciences approved all procedures performed in the current study with the approval number: IR.TUMS.SHARIATI.REC.1402.055.

## Conflicts of Interest

The authors declare no conflicts of interest.

## Data Availability

The data that support the findings of this study are available from the corresponding author, upon reasonable request.
